# Starch as a Matrix for Incorporation and Release of Bioactive Compounds: Fundamentals and Applications

**DOI:** 10.3390/polym14122361

**Published:** 2022-06-10

**Authors:** Lucas de Souza Falcão, Deborah Bento Coelho, Priscilla Carvalho Veggi, Pedro Henrique Campelo, Patrícia Melchionna Albuquerque, Mariana Agostini de Moraes

**Affiliations:** 1 Department of Chemical Engineering, Federal University of Sao Paulo, UNIFESP, Diadema 09913-030, SP, Brazil; lsfalcao@unifesp.br (L.d.S.F.); deborahdscoelho@gmail.com (D.B.C.); pveggi@unifesp.br (P.C.V.); 2 Department of Food Technology, Federal University of Viçosa, Viçosa 36570-900, MG, Brazil; pedrocampelo@ufv.br; 3 School of Technology, Amazonas State University, UEA, Manaus 69065-020, AM, Brazil; palbuquerque77@gmail.com

**Keywords:** biopolymer, natural extract, starch

## Abstract

Due to its abundance in nature and low cost, starch is one of the most relevant raw materials for replacing synthetic polymers in a number of applications. It is generally regarded as non-toxic, biocompatible, and biodegradable and, therefore, a safe option for biomedical, food, and packaging applications. In this review, we focused on studies that report the use of starch as a matrix for stabilization, incorporation, or release of bioactive compounds, and explore a wide range of applications of starch-based materials. One of the key application areas for bioactive compounds incorporated in starch matrices is the pharmaceutical industry, especially in orally disintegrating films. The packaging industry has also shown great interest in using starch films, especially those with antioxidant activity. Regarding food technology, starch can be used as a stabilizer in nanoemulsions, thus allowing the incorporation of bioactive compounds in a variety of food types. Starch also presents potential in the cosmetic industry as a delivery system. However, there are still several types of industry that could benefit from the incorporation of starch matrices with bioactive compounds, which are described in this review. In addition, the use of microbial bioactive compounds in starch matrices represents an almost unexplored field still to be investigated.

## 1. Introduction

In the last decades, increased concern regarding environmental impact related to human activity has been expressed worldwide. Plastics have been extensively used in many applications, from packaging to the medical industry, generating large amounts of non-degradable solid waste, which are considered one of the main environmental problems of today.

In this context, biopolymers emerge as an alternative to synthetic polymers, and can help to reduce the environmental impact caused by the latter. Moreover, biopolymers are promising candidates for materials used in biotechnology applications because they tend to be compatible with diverse types of cells and tissues. In addition, they can be modified in a wide range of manners, which raises their applicability, in addition to being biodegradable and originating from renewable materials [1]. Among the natural polymers that have biotechnological potential, starch can be highlighted, particularly for its physical, chemical, and biological characteristics. It has good film-forming abilities [2,3], and tends to form thin and transparent films, without color or odor, and presents efficient CO_2_ and O_2_ barrier characteristics, thus displaying the capacity to protect food products [4] that could be enhanced by the addition of bioactive molecules [5]. However, starch-based materials may present weak mechanical properties and poor long-term stability, though this could be improved via chemical or physical modifications in the starch matrix. Bioactive molecules can benefit from being incorporated to starch-based matrices since it is reported that polysaccharide microparticles, for example, tend to enhance systemic absorption of drugs when compared to traditional formulations, as well as lowering the frequency required for the dose [6,7]. Moreover, the incorporation of bioactive molecules in starch-based matrices can improve their bioactive stability, thereby increasing their range of applicability [2,3].

The association of biopolymers and bioactive compounds has been extensively studied and has resulted in the development of bioactive materials with improved properties, such as antioxidant [8] and antimicrobial properties [9,10,11], which are important components for biotechnological applications in the food, pharmaceutical, nutraceutical, and cosmetic industries. Usually, bioactive compounds are added to enhance the characteristic properties of these materials, such as oxidation resistance, antimi crobial activity, and mechanical and barrier properties [12].

**Figure 1:**
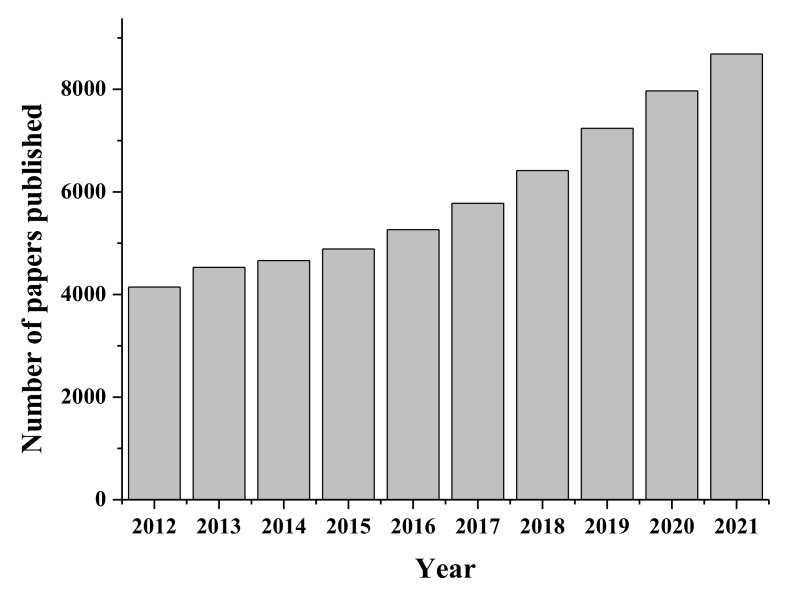
Number of papers published per year in the last decade with the keyword “starch”. Data extracted from the Web of Science database on 3 June 2022.

Starch is one of the most abundant biopolymers in the world and is greatly explored because of its abundance and susceptibility to physical and chemical modifications, including its capacity to form thermoplastics [13,14]. The importance of starch and the increase in research regarding its applications can be witnessed by analyzing the publications in the last decade. Figure 1 presents the number of publications by year from 2012 to 2021 and shows that the number of studies published on starch doubled, from 4145 to 8688 publications, which indicates the increasing interest in starch-related research.

In order to select the papers that were analyzed in this review, the results from the Web of Science database pertaining to starch-related research were refined by including the terms “bioactive compounds” or “natural extract”, which resulted in 98 papers. Then, from these papers, the ones that used starch as a matrix for incorporating or releasing extracts or bioactive compounds were selected.

## 2. Starch

Starch is the main carbohydrate that functions as an energy store in plants and is a significant nutritional source for animals. It is a polysaccharide that is composed of two polymers, amylose, a linear polymer, and amylopectin, and has highly branched chains [15]. Amylose is a mainly linear polymer that consists of α (1,4) linked D-glucopyranosyl units, while amylopectin is a branched polymer of α-D-glucopyranosyl units that are primarily linked by (1,4) bonds with branches resulting from (1,6) linked D-glucopyranosyl units [16].

Different sources of starches tend to have variations in the proportions of amylose and amylopectin and, as a consequence, the resulting product can present different characteristics. A film, for example, may present more flexibility as a result of a higher amount of amylose; therefore, the use of new starch sources presents advantages due to the versatility of starch, and creates numerous forms for the incorporation of bioactive compounds for different applications [17,18].

Two main starch components, amylose and amylopectin, have different contributions to the supramolecular structure of starch. Single helical chains of amylose disrupt the structural order and lead to the formation of amorphous domains in starch, while double helices of amylopectin form highly ordered crystalline domains of starch. The combination of amorphous and crystalline domains results in the formation of three main types of semi-crystalline starch granules, such as type A (found in cereals) [19], type B (common in tubercles, cladodes, and fruits) [20], and type C (generally found in legumes) [21].

Starch extraction is based primarily on the cellular rupture of the plant tissue, liberating the starch granules to a solvent (usually water) [22]. Depending on the interaction between the starch granule and the plant tissue, physical and auxiliary chemical methods may be used to enhance the yield of the extraction [23]. Tagliapietra et al. [24] described a protocol of extraction of new types of starch and the principal parameters to diminish any damage to the starch structure. They observed that the type of extraction can also affect the amylose-amylopectin proportion of starch, further increasing the versatility of this biopolymer [18].

An important process in starch applications is gelatinization, which occurs when starch undergoes a structural change after being heated above a certain temperature, normally ranging from 60 °C to 70 °C. This leads to the formation of hydrogen bonds between hydroxyl groups of starch and water in a way that, at a determined concentration, water acts as a plasticizer of the amorphous parts of the starch granule. Another process that must be taken into account in the application of starch in industrial processes is retrogradation, in which the starch molecule recovers parts of its crystalline structure. It is important to note that the amylose retrogradation happens in a much shorter time than amylopectin retrogradation; the former can occur in 48 h, while the latter tends to occur in 30 to 40 days [25].

In its native form, starch may not present interesting properties to the industry since it has high water solubility and swelling power [26], low gelatinization temperature and tendency to suffer retrogradation, in addition to having low tensile strength [27]. However, starch can be modified through physical methods (heat-humidity treatment, annealing, retrogradation, pre-gelatinization, and high pressure) [28], chemical methods (reticulation, esterification, acid treatment, and oxidation) [29], enzymatic modification (using amylolytic enzymes), or genetic mutation, in order to improve its properties for a wide range of industrial applications [30,31].

**Figure 2:**
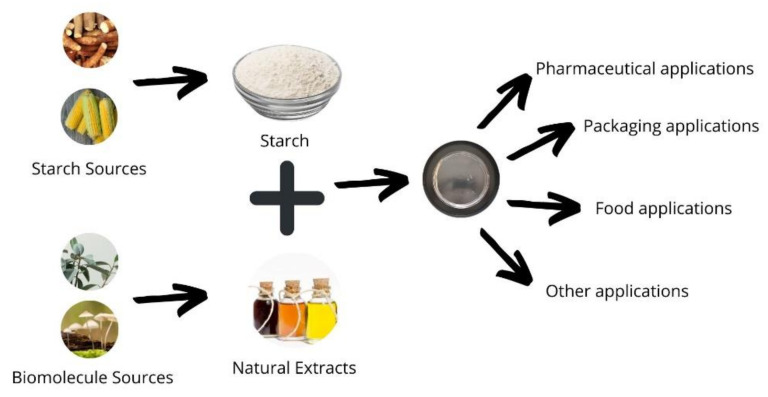
Schematic representation of starch and sources of natural extracts and potential applications. Figures obtained from canva.com (accessed on 25 April 2022).

In general, starch presents good properties for film formation [32,33,34,35], particulate systems [36,37], and gel formation [38,39,40,41,42], and is adequate for incorporating bioactive substances in controlled-release devices. An example of the process and its applications can be seen in Figure 2.

Depending on the molecular structure of the bioactive, the bioactive compounds can be physically or chemically incorporated into starch. Considering that starch matrices have high swelling capability, the extracts or bioactive compounds can be physically located in the intermolecular structure of starch, and can also act as a plasticizer in starch films [43]. In addition, starch has hydroxyl functional groups that can chemically interact with the molecular structure of the bioactive compounds. The main possible interactions between starch and bioactive compounds are electrostatic interactions and hydrogen bonding [18].

## 3. Bioactive Compounds

Bioactive compounds have been extensively used around the world and, in recent years, have attracted the attention of scientists since their consumption has been connected with healthier habits, in order to prevent and treat various diseases [44]. Initially, plants were used in the form of tinctures, teas, poultices, and powders, among other types of pharmaceutical formulations, in an empirical way. Later, a solid scientific basis was added to the traditional knowledge, which resulted in the development of new drugs from extracts and oils. It was then possible to identify and isolate the bioactive substances of interest [45].

The knowledge and applications of bioactive compounds are well-known and widely applied in different segments such as the pharmaceutical, food, and chemical industries. Bioactive compounds are commonly found in a large number of organisms, from which they can be isolated using extraction and biotechnological methods [46]. Several of the recently launched pharmaceuticals are derived from plants and microorganisms isolated from different sources [47].

Metabolites produced by plants and microorganisms are classified as either primary or secondary metabolites. Primary metabolites provide essential substances for cellular homeostasis, such as sugars, amino acids, nucleotides, organic acids, and fatty acids [48]. Secondary metabolites are essential in the interaction, adaptation, and survival of these organisms. They occur in low concentrations and specific cells and may be bioactive substances that exert pharmacological and/or toxicological effects in humans or animals [49].

Secondary metabolites are formed by a wide variety of molecules; some are more restricted and are only present within a certain species or taxonomic group [50]. However, with the evolution of methodologies for the elucidation of chemical substances, it has been possible to characterize the substances that are present even in small concentrations, thus demonstrating the diversity and chemical complexity synthesized by plants and microorganisms. These metabolites are formed according to the coevolution of plants, insects, microorganisms, and mammals, which in turn leads to the synthesis of metabolites with defense or attraction functions [51].

A large number of plants and microorganisms have been recognized as valuable sources of natural substances, since they are rich in a variety of molecules that are synthesized in their secondary metabolism, such as vitamins, polyphenols, terpenoids, flavonoids, carotenoids, essential oils, and proteins, among others. The diversity of bioactive compounds in plants varies according to the part of the plant, such as leaves, bark, roots, flowers, seeds [52,53], or may be found in the fruit or vegetables produced by the plant [54].

Secondary metabolites are reported in the literature as having a wide range of biological properties, such as antimicrobial [55], antioxidant, antitumor, antiparasitic, and anti-inflammatory properties, among others, and thus become important therapeutic resources for human health [56,57]. Furthermore, according to Newman and Cragg [58], natural products are the best options for obtaining new substances that can lead to efficient agents against various diseases, especially against bacterial and fungal infections and cancer.

The interest in bioactive substances of natural origin has grown over the years. The development of bioproducts, thus, depends on natural active substances, and these are often unstable compounds, which can cause reactions that promote a decrease or loss of efficiency and even the degradation of the product. Consequently, new technologies have been proposed to improve the performance of pharmaceutical, food, and cosmetic products produced from natural raw materials, which favors their acceptance by the consumer. An alternative that increases stability and allows the controlled release of natural substances is the encapsulation technique using polymeric matrices. The compartmentalization of bioactive substances in polymeric carriers with specific physicochemical properties such as starch is, therefore, an interesting alternative for increasing the use of natural bioactive compounds in different applications, as can be observed through the wide range of applications presented in this review and summarized here in Table 1.

## 4. Pharmaceutical Applications

Controlled-release devices appear as an alternative to traditional drug administration methods, which usually present low efficacy and the necessity for large dosages [98]. They may present different forms, such as gels, hydrogels, nanoparticles, and films [99]. The inclusion of bioactive molecules in biopolymeric matrices allows us to direct the bioactive to a specific site, promoting local and controlled release, and prolonging the molecule’s action directly on the desired tissue [100].

Since 2017, starch has been used in various forms as a carrier for the release of different drugs. Panyoyai, Shanks, and Kasapis [59] showed that microcapsules made from waxy maize starch can be used to release tocopheryl acetate. Modified starches can provide multiple possibilities for delivery systems. The studies by Jung et al. [60] showed that glucan-coated porous starch granules can be used as an encapsulant for crocin, which directly enhanced the retention of the biomolecule in the starch granules.

One strong trend in delivery systems using starch is the encapsulation of curcumin. For this purpose, Athira, Jyothi, and Vishnu [61] developed water-soluble nanoparticles of octenyl succinylated cassava starch loaded with curcumin aiming to enhance bioavailability and anticancer potential. Similar research was developed by Luo, Adra, and Kim [62], who encapsulated a curcumin-cyclodextrin complex into microparticles made from enzymatic-modified waxy maize starch to improve the stability of curcumin against chemical oxidation and photodegradation, and also raise its bioavailability via control of the release rate and better stabilization in gastric conditions. In addition, Lu, Li, and Huang [63] encapsulated curcumin into Pickering emulsions stabilized with starch particles, which enhanced the stability of the curcumin under simulated gastric conditions.

Incorporation of turmeric in films based on starch and chitosan was performed by Schaefer et al. [64]. Although the films containing only chitosan presented antimicrobial activity and adhesion to pork mucosa, the films blended with starch showed themselves to be more adequate for biomedical applications, as it diminishes the solubility of the films.

Other studies also should be mentioned. Oliyaei et al. [65] produced fucoxanthin encapsulated with porous starch, which exhibited therapeutic efficacy in a type 2 diabetic mice model. Costa et al. [66] developed films composed of blends of polyvinyl alcohol, starch and polyacrylic acid with the incorporation of pomegranate peel extract, which showed positive results in wound healing tests (in vitro).

Starch has also been used in the formulation of orally disintegrating films, which are alternatives to traditional methods of drug administration, especially because they have the potential for fast release and are absorbed directly into the oral mucosa without the need for ingestion of water. In this context, special attention has been given to biopolymers due to their hydrophilicity, non-toxicity, and mucoadhesion properties [101].

Starch-based orally disintegrating films have a remarkable ability to deliver molecules with pharmaceutical potential. Garcia et al. [67] produced starch and gelatin-based films and incorporated acerola (*Malpighia emarginata*) extract, which contains high levels of vitamin C. The authors observed that in films with higher concentrations of starch, vitamin C presented greater stability when compared to those with higher concentrations of gelatin, thus demonstrating the potential of starch as a film matrix for natural compounds. A similar study was performed with camu-camu (*Myrciaria dubia*) extract [68], in which a higher concentration of starch promoted higher hydrophilicity, raising the degradation time of the orally disintegrating film, which could be interesting for several applications.

Guerra et al. [69] included mango peel extract in the starch to use the final film as a carrier of phenolic compounds. The authors considered that the films based on corn starch maintained the antioxidant activity since the incorporated phenolic compounds did not suffer significant oxidative degradation. Bodini et al. [70] also developed orally disintegrating films based on starch and hydroxypropyl methylcellulose and containing an incorporated extract of *Cordia verbenacea*, which successfully retained anti-inflammatory and antioxidant activity. In addition, the films presented a higher concentration of flavonoids.

As can be seen, by being non-toxic and biocompatible with various natural compounds, starch-based materials are potential carriers of bioactive compounds, especially in the form of orally disintegrating films.

## 5. Packaging Applications

Synthetic polymers have been the main component used in the packaging industry in the last decades. Despite their remarkable characteristics, such as good mechanical properties and relatively low production cost, they originate from fossil resources and generate a large volume of solid residues [102]. In this context, the packaging industry has started to search for less impactful options to replace plastic [103,104]. The main candidates for solving the problem of the non-biodegradable residues generated by the packaging industry are biopolymers. Since they are degraded by naturally occurring microorganisms, they can be degraded at the disposal site under natural conditions. For this reason, biopolymers have attracted the attention of the packaging industry in the last decade [105].

To improve the properties of a film intended to be used in the packaging industry, a wide variety of natural compounds can be used, such as adding antimicrobial or antioxidant properties to the films. One of the first studies in this area was done by Pelissari et al. [71]. They prepared flexible starch-chitosan films containing incorporated oregano essential oil, which demonstrated antimicrobial activity. However, starch-based films with incorporated natural extracts and active packaging properties had not received much attention until the second half of the last decade. Araújo et al. [72] produced cassava starch-based films by casting and incorporated an ethanolic propolis extract in the carrier. The films had the ability to release phenolic compounds with antioxidant activity and showed antimicrobial activity against the pathogenic bacteria *Staphylococcus aureus* and *Escherichia coli*.

Araújo et al. [9] produced a cassava starch-chitosan edible coat incorporated with the essential oil of *Lippia sidoides* cham. and pomegranate peel extracted at different concentrations and tested its effects on the quality of tomatoes. The results showed that starch is a potential material for food packaging by lowering the weight loss when compared to the control group of uncoated tomatoes.

Caetano et al. [10] developed cassava starch biodegradable films incorporated with essential oils of oregano and pumpkin peel extract. The films produced were tested regarding lipid oxidation of ground beef and protected the product until the third day of the analysis. Furthermore, the films incorporated with the highest concentrations of oregano essential oil presented antimicrobial activity in the tests, thus demonstrating the potential of starch-based films and the natural compounds utilized in this study on meat preservation. In a similar study, Taweechat et al. [73] obtained promising results for the conservation of food by using films produced with banana starch incorporated with banana peel extract in the conservation of minced pork. Boeira et al. [74] developed a film based on corn starch and extract of corn stigma that demonstrated antioxidant properties and reduced lipid oxidation by 60%. In addition, the film showed antimicrobial activity against mesophilic and psychrotrophic bacteria.

Malherbi et al. [75] produced biodegradable films based on a blend of gelatin and corn starch incorporated with gabiroba (*Campomanesia xanthocarpa*) pulp, a natural antioxidant, and analyzed its application in the storage of extra virgin olive oil. They noted that the stored product was within the Brazilian parameters for the studied period (15 days). Cheng et al. [5] obtained an improvement in the light and oxygen barrier properties of the films incorporated with red cabbage anthocyanin extract, as well as antioxidant properties, which could improve the shelf life of the product. In the work of Tongdeesoontorn et al. [76], films composed of starch and gelatin were able to postpone the oxidation of lard, besides delaying the discoloration of the product. This occurred when using quercetin and thertiary butylhydroquinone (TBHQ) as antioxidants.

Peralta et al. [77] used an aqueous hibiscus extract incorporated in films based on three different natural polymers (chitosan, gelatin, and starch) to produce a pH-sensitive indicator based on the stability of the pigment, and obtained a visible and pH-dependent variation in color for all polymeric films. The gelatin film had the best color variation. Nevertheless, starch films presented a similar variation in color to gelatin, which represents the potential of the films produced as a potential pH indicator in biopolymeric films.

Another natural extract with the potential to be incorporated into biopolymeric films is the *Araucaria angustifolia* extract, as demonstrated by Da Silva et al. [78], which was incorporated into films based on cassava starch and poly (butylene adipate co-terephthalate), also known as PBAT. Additionally, Muller et al. [79] demonstrated that cassava starch could be replaced by *A. angustifolia* starch, which was extracted from its seeds. The authors also successfully incorporated rosemary and green tea aqueous extract in the films, and the extract directly influenced the physicochemical properties of the produced films.

An interesting approach to the development of active packaging materials is the incorporation of antibacterial molecules into the biopolymer. An example of this case can be seen in the research performed by De Oliveira et al. [80] who developed a composite film based on starch and polyvinyl alcohol with different concentrations of maleic acid, cellulose nanocrystals, and nisin Z, which showed activity against *Listeria monocytogenes*.

Cunha et al. [11] investigated the production of cassava starch-based films incorporated with propolis extract that resulted in a more flexible film with antioxidant properties, which are characteristics that may be desired for certain packaging objectives. Similar results were also obtained by Farias et al. [8] with the use of nopal cladode flour to reinforce starch-based films. This provided the films with antioxidant activity, and showed a shift on FTIR spectra in the region associated with hydroxyl groups, thus indicating the importance of this functional group in the bonding between the starch matrix and the bioactive compounds. Da Silva, Velasco, and Fakhouri [33] demonstrated that, depending on the starch source, the film can demonstrate antioxidant properties itself, since films produced from different types of rice showed the presence of phenolic compounds.

Therefore, the natural properties of starch films, allied to the potential of the active compounds’ biodiversity, show that the packaging industry can take advantage of these products while reducing the amount of non-biodegradable residues generated, and improving the functions of the food-packaging.

## 6. Food Applications

Starch can also be used as a raw material to encapsulate compounds with interesting properties for food applications, especially molecules that are sensitive to oxidation or degradation under different conditions, and which diminishes the product’s final quality. Encapsulation techniques, such as spray drying, extrusion, and emulsification can be used with starch to encapsulate the desired compound [106].

Souza et al. [81] used starch as an adjuvant for spray drying to encapsulate pigments produced by fungi isolated from caves in Brazil, which leads to high pigment retention and could improve the stability of the compound during storage. Ribeiro et al. [82] used blends of starch, gum arabic, and maltodextrin to encapsulate vitamin A, and observed that the presence of starch in the blend led to a slower release rate of the molecule. Tomsone et al. [83] also used starch, in addition to other biopolymers, as a core material in spray-drying of horseradish juice, and obtained larger and less soluble particles using starch.

Starch has also been used as a stabilizer in nanoemulsions, as shown by Abbas et al. [84]. A curcumin oil-water nanoemulsion was formed, and octenyl succinic anhydride-modified starch, an amphiphilic polysaccharide, was used as a stabilizer for the emulsion. Octenyl-modified starch was also used as an emulsifier by Doost et al. [85] to encapsulate lutein in order to produce a nutraceutical beverage, and by Niu et al. [86], who produced a nanoemulsion loaded with coenzyme Q10 using the same modified starch as a stabilizer. The octenyl-modified starch was more stable than the other agents tested (whey protein isolate and lecithin) in gastric digestion conditions. Furthermore, nanoemulsions were also prepared by Hasani, Ojagh, and Ghorbani [87], who used microparticles of chitosan and modified starch to encapsulate essential oil from lemon, which is a compound that is sensitive to adverse storage conditions. The encapsulation of the two biopolymers demonstrated their capacity to protect the bioactive compound from volatilization, thus increasing the stability and increasing the bioactive potential to be used as a food additive.

Another method that can be used to encapsulate compounds in biopolymers is freeze-drying, as shown in Silva et al. [88]. The authors encapsulated tucumã (*Astrocaryum aculeatum*) pulp with various biopolymers and observed that the modified starch was the one with best bioactive compound retention. Similar work was performed by Ravichai and Muangrat [89], who used different biopolymers to encapsulate phenolic compounds derived from fermented Miang wastewater by freeze-drying, and the authors selected gum arabic as the best coating material for encapsulating the highest amount of phenolic compounds. Even so, it is important to note that the Miang powder encapsulated with modified starch maintained its antioxidant properties.

Zheng et al. [90] produced a yogurt containing double polysaccharide film-coated microparticles to release of thymopoietin. The two different coatings, composed of chitosan and modified starch, allowed the bioactive compound to be delivered to the colon, maximizing its biological activity. In the work of Wang et al. [91], the authors used octenyl succinate starch as a carrier for the delivery of bioactive food components, successfully using 5-aminosalicylic acid and bovine serum albumin as a model and demonstrated the potential of this type of modified starch as a delivery carrier.

Natural extracts can also be used to supplement starch-based foods, as is the case of Rodrigues et al. [92], who used a propolis co-product in the supplementation of a starch biscuit based on canola oil, which prevented lipid oxidation.

Considering the studies in the literature regarding the use of starch with natural extracts, it becomes clear that there is great potential for developing functional foods that could be used as raw material and enhance the quality of a wide range of products. Moreover, these functional foods would bring several health benefits to consumers.

## 7. Other Applications

Biopolymers are already broadly used in cosmetic formulations, especially as moisturizers and thickeners; starch can form hydrogels with cross-linked networks that tend to have high water absorption capacity [107]. Therefore, starch matrices, incorporated with natural extracts, have been studied for cosmetic purposes. Daudt et al. [93] used pinhão derivatives, starch and coat extract, as raw materials for cosmetics. The authors found that the pinhão derivatives are sources of phenolic compounds with antioxidant activity.

Silveira et al. [94] developed films based on starch and cellulose nanofiber that were incorporated with tea tree essential oil and investigated their antimicrobial applications. The authors observed that the film could inhibit the growth of the Gram-positive bacteria *Sthaphylococcus aureus* and the yeast *Candida albicans*, thus showing that the biopolymers present potential as a matrix for the release of natural compounds, and resulting in a product with a diverse range of applications. An interesting study was developed by Wu et al. [95], who used electrospun starch-derived nanographene oxide as scaffolds for bone tissue engineering.

Hornung et al. [96] produced starch-based films incorporated with extract of yerba mate and demonstrated that the films maintained antioxidant activity and total phenolic and total flavonoids content, as well as other desirable properties such as lower moisture content, solubility, and water vapor permeability. In addition, this work demonstrated how the incorporation of extracts in the film can affect its physical properties, lowering the degree of crystallinity, and suggesting that the extract can act as a plasticizer by increasing the amorphous region in starch.

Another study that examined the use of starch as a matrix in cosmetic formulations was developed by Pereira, Lonny, and Mali [97], who incorporated catuaba (*Trichilia catigua*) extract in starch films, and the films loaded with the highest concentration of catuaba extract showed promising results for use as a delivery system in cosmetic formulations.

## 8. Conclusions

Therefore, as the need for substitution of plastic material increases, being a biopolymer with good availability, non-toxicity, and biodegradability, starch appears to be an alternative and is a good candidate for use as a matrix in a wide range of applications, especially when incorporated with bioactive compounds. The incorporation of bioactive compounds in biopolymeric matrices can enhance the bioactive stability, one of the main points of concern in the use of certain types of bioactive compounds, allowing these devices to be used in applications in pharmaceutical products, packaging, food technology, and others. As an example, it is possible to cite the incorporation of curcumin in starch matrices [61,62,63]. Orally disintegrating films based on starch have also demonstrated good potential for incorporating bioactive compounds, especially when blended with other biopolymers [67,68].

In the packaging industry, bioactive compounds that add antioxidant activity to the films and coatings are the main targets [10]. In addition, molecules with antimicrobial activities also have good potential to be used, especially in active packaging [80].

Starch has also been well studied as an adjuvant for spray drying and as a stabilizer for nanoemulsions [82,86], demonstrating good applicability in the food industry [90].

Just a few studies in the literature have explored starch-based matrices for cosmetic applications [97], making this area one of the most interesting trends in the utilization of starch for incorporating bioactive compounds.

In this review, we highlighted the potential use of starch as matrix for incorporation and release of a wide variety of bioactive compounds, which enhances its stability and increases its range of application.

## Figures and Tables

**Table 1:** Summary of starch matrices incorporated with bioactive compounds.

Nº	Starch Source	Starch Matrix	Bioactive Source	Bioactive	Properties	Application
[59]	Waxy maize starch	Microcapsules	Commercially obtained	Tocopheryl acetate	Controlled release	Pharmaceutical
[60]	Porous corn starch	Coated granules	Commercially obtained	Crocin	Controlled release	Pharmaceutical
[61]	Succinylated cassava starch	Nanoformulation	Commercially obtained	Curcumin	Anticancer	Pharmaceutical
[62]	Waxy maize starch	Microparticles	Commercially obtained	Curcumin	Enhanced stability	Pharmaceutical
[63]	Maize starch	Particle	Commercially obtained	Curcumin	Enhanced stability	Pharmaceutical
[64]	Corn starch and chitosan	Film	Commercially obtained	Turmeric	Antimicrobial	Pharmaceutical
[65]	Porous corn starch	Encapsulation	*Sargassum angustifolium*	Fucoxanthin	Antidiabetic effect	Pharmaceutical
[66]	Blended polyvinyl alcohol, potato starch and polyacrylic acid	Film	Pomegranate peel extract	-	Wound healing	Pharmaceutical
[67]	Gelatin and pregelatinized cassava starch	Film	Acerola (*Malpighia emarginata*)	Vitamin C	Enhanced stability	Pharmaceutical
[68]	Gelatin and pregelatinized modified cassava starch	Film	Camu-camu (*Myrciaria dubia)*	Vitamin C	Enhanced stability	Pharmaceutical
[69]	Corn starch	Film	*Mangifera indica*	Phenolic compounds	Antioxidant	Pharmaceutical
[70]	Pregelatinized modified cassava Starch	Film	*Cordia verbenacea*	Flavonoids	Antioxidant and anti-inflammatory	Pharmaceutical
[71]	Cassava starch and chitosan	Film	Oregano essential oil	-	antimicrobial	Packaging
[72]	Cassava starch	Film	Propolis	Phenolic compounds	Antimicrobial and Antioxidant	Packaging
[9]	Cassava starch–chitosan	Edible coating	*Lippia sidoides* essential oil and pomegranate peel extract	-	Food preservation	Packaging
[10]	Cassava starch	Film	Oregano essential oil and pumpkin peel extract	-	Antioxidant and antimicrobial	Packaging
[73]	Banana starch	Film	Banana peel extract	-	Food conservation	Packaging
[74]	Corn starch	Film	Corn stigma extract	-	Antioxidant and antimicrobial	Packaging
[75]	Corn starch and gelatin	Film	Guabiroba pulp (*Campomanesia xanthocarpa*)	-	antioxidant	Packaging
[5]	Modified Starch	Film	Red cabbage	Anthocyanin	Light and oxygen barrier	Packaging
[76]	Cassava Starch and gelatin	Film	-	Quercetin and tertiary butylhydroquinone	Antioxidant	Packaging
[77]	Modified cassava starch	Film	Hibiscus extract	-	pH Indicator	Packaging
[78]	Cassava starch and poly (butylene adipate co-terephthalate)	Film	*Araucaria angustifolia*	Phenolic compounds	Antioxidant	Packaging
[79]	Pine nut and cassava starch	Thermoplastic	Rosemary and green tea aqueous extract	-	Modification of physico-chemical properties	Packaging
[80]	Corn starch and polyvinyl alcohol	Film	Commercially obtained	Nisin Z	Antimicrobial	Packaging
[11]	Cassava starch	Film	Propolis extract	-	antioxidant	Packaging
[8]	Cassava starch	Film	Nopal cladode flour	-	Antioxidant	Packaging
[33]	Rice starch	Film	Rice	Phenolic compounds	Antioxidant	Packaging
[81]	Modified starch	Adjuvant for spray drying	Fungi isolated from Brazilian caves	Pigments	Enhanced stability	Food technology
[82]	Blends of gum arabic, starch and maltodextrin	Microencapsulation	Commercially obtained	Vitamin A	Enhanced stability	Food technology
[83]	Soluble starch	Core material in spray-drying	Horseradish juice	Phenolic compounds	Enhanced stability	Food technology
[84]	Octenyl succinic anhydride modified starch	Nanoemulsion stabilizer	Commercially obtained	Curcumin	Enhanced stability	Food technology
[85]	Octenyl succinic anhydride modified starch	Nanoemulsion stabilizer	Commercially obtained	Lutein	Enhanced stability	Food technology
[86]	Octenyl succinic anhydride modified starch	Nanoemulsion stabilizer	Commercially obtained	Coenzyme Q10	Enhanced stability	Food technology
[87]	Chitosan and modified starch	Nanoencapsulation	Lemon essential oil	-	Enhanced stability	Food technology
[88]	Modified starch	Encapsulation	Tucumã powder (*Astrocaryum aculeatum*)	-	Enhanced stability	Food technology
[89]	Modified starch	Coating material in freeze-drying	Fermented tea leaf wastewater	Phenolic compounds	antioxidant activity	Food technology
[90]	Chitosan and modified starch	Film-coated microparticles	Commercially obtained	Thymopoietin	Controlled release	Food technology
[91]	Octenyl succinate starch	Delivery carrier	-	-	-	Food technology
[92]	Starch biscuit	-	Propolis co-product extract	-	Antioxidant	Food technology
[93]	Pine nut starch	Topical formulation	Pine nut skins	Phenolic compound	Antioxidant	Cosmetic
[94]	Cassava starch and cellulose	Nanofiber film	Tea tree essential oil	-	Antimicrobial	Several applications
[95]	Maize starch	Starch/nano graphene oxide nanofibers	-	-	-	Bone tissue engineering
[96]	Corn starch	Film	*Ilex paraguariensis*	Phenolic compounds	Antioxidant	Several applications
[97]	Cassava starch	Film	Catuaba extract (*Trichilia catigua*)	Vitamin C	Antioxidant	Cosmetic

## Data Availability

Not applicable.

## References

[1] Joye I.J., McClements D.J. (2015). Biopolymer-Based Delivery Systems: Challenges and Opportunities. Curr. Top. Med. Chem..

[2] Builders P.F., Arhewoh M.I. (2016). Pharmaceutical applications of native starch in conventional drug delivery. Starch-Stärke.

[3] Ogunsona E., Ojogbo E., Mekonnen T. (2018). Advanced Material Applications of Starch and Its Derivatives. Eur. Polym. J..

[4] Thakur R., Pristijono P., Scarlett C.J., Bowyer M., Singh S.P., Vuong Q.V. (2019). Starch-based films: Major factors affecting their properties. Int. J. Biol. Macromol..

[5] Cheng M., Yan X., Cui Y., Han M., Wang Y., Wang J., Zhang R., Wang X. (2022). Characterization and Release Kinetics Study of Active Packaging Films Based on Modified Starch and Red Cabbage Anthocyanin Extract. Polymers.

[6] Xie F., Pollet E., Halley P.J., Avérous L. (2013). Starch-Based Nano-Biocomposites. Prog. Polym. Sci..

[7] Rodrigues A., Emeje M. (2012). Recent applications of starch derivatives in nanodrug delivery. Carbohydr. Polym..

[8] de Farias P.M., de Vasconcelos L.B., Ferreira M.E.D.S., Filho E.G.A., De Freitas V.A., Tapia-Blácido D.R. (2021). Nopal cladode as a novel reinforcing and antioxidant agent for starch-based films: A comparison with lignin and propolis extract. Int. J. Biol. Macromol..

[9] Araújo J.M.S., de Siqueira A.C.P., Blank A.F., Narain N., de Aquino Santana L.C.L. (2018). A Cassava Starch–Chitosan Edible Coating Enriched with Lippia sidoides Cham. Essential Oil and Pomegranate Peel Extract for Preservation of Italian Tomatoes (Lycopersicon esculentum Mill.) Stored at Room Temperature. Food Bioprocess. Technol..

[10] Dos Santos Caetano K., Almeida Lopes N., Haas Costa T.M., Brandelli A., Rodrigues E., Hickmann Flôres S., Cladera-Olivera F. (2018). Characterization of active biodegradable films based on cassava starch and natural compounds. Food Packag. Shelf Life.

[11] Cunha G.F., Soares J.C., de Sousa T.L., Egea M.B., de Alencar S.M., Belisário C.M., Plácido G.R. (2021). Cassava-Starch-Based Films Supplemented with Propolis Extract: Physical, Chemical, and Microstructure Characterization. Biointerface Res. Appl. Chem..

[12] Rangaraj V.M., Rambabu K., Banat F., Mittal V. (2021). Natural Antioxidants-Based Edible Active Food Packaging: An Overview of Current Advancements. Food Biosci..

[13] Torres F.G., Commeaux S., Troncoso O.P. (2013). Starch-based biomaterials for wound-dressing applications. Starch-Stärke.

[14] Fan Y., Picchioni F. (2020). Modification of Starch: A Review on the Application of “Green” Solvents and Controlled Functionali-zation. Carbohydr. Polym..

[15] Whistler R.L., Daniel J.R. (2000). Starch. Kirk-Othmer Encycl. Chem. Technol..

[16] Faisal M., Kou T., Zhong Y., Blennow A. (2022). High Amylose-Based Bio Composites: Structures, Functions and Applications. Polymers.

[17] Tharanathan R.N. (2003). Biodegradable films and composite coatings: Past, present and future. Trends Food Sci. Technol..

[18] Nogueira G.F., Oliveira R.A., Velasco J.I., Fakhouri F.M. (2020). Methods of incorporating plant-derived bioactive compounds into films made with agro-based polymers for application as food packaging: A brief review. Polymers.

[19] Buleon A., Trm V., Mrez S. (1991). Recent Advances in Knowledge of Starch Structure. Starch-Stärke.

[20] Imberty A., Perez S. (1988). A revisit to the three-dimensional structure of B-type starch. Biopolymers.

[21] Pinto C.D.C., Sanches E.A., Clerici M.T.P.S., Pereira M.T., Campelo P.H., de Souza S.M. (2021). X-ray diffraction and Rietveld characterization of radiation-induced physicochemical changes in Ariá (Goeppertia allouia) C-type starch. Food Hydrocoll..

[22] Cereda M., Sarmento S., Vilpoux O. (2005). Effects of Extraction Methods on Yam (Dioscorea alata) Starch Characteristics. Starch-Stärke.

[23] Kringel D.H., El Halal S.L.M., Zavareze E.D.R., Dias A.R.G. (2020). Methods for the Extraction of Roots, Tubers, Pulses, Pseudocereals, and Other Unconventional Starches Sources: A Review. Starch-Stärke.

[24] Tagliapietra B.L., Felisberto M.H.F., Sanches E.A., Campelo P.H., Clerici M.T.P.S. (2021). Non-Conventional Starch Sources. Curr. Opin. Food Sci..

[25] Ledezma C.C.Q. (2018). Starch Interactions with Native and Added Food Components.

[26] Carvalho A.P.M.G., Barros D.R., da Silva L.S., Sanches E.A., Pinto C.D.C., de Souza S.M., Clerici M.T.P.S., Rodrigues S., Fernandes F.A.N., Campelo P.H. (2021). Dielectric barrier atmospheric cold plasma applied to the modification of Ariá (*Goeppertia allouia*) starch: Effect of plasma generation voltage. Int. J. Biol. Macromol..

[27] Ashogbon A.O. (2020). The Recent Development in the Syntheses, Properties, and Applications of Triple Modification of Various Starches. Starch-Stärke.

[28] Grgić I., Ačkar Đ., Barišić V., Vlainić M., Knežević N., Medverec Knežević Z. (2019). Nonthermal Methods for Starch Modifica-tion—A Review. J. Food Process. Preserv..

[29] Masina N., Choonara Y.E., Kumar P., du Toit L.C., Govender M., Indermun S., Pillay V. (2017). A Review of the Chemical Mod-ification Techniques of Starch. Carbohydr. Polym..

[30] Kaur B., Ariffin F., Bhat R., Karim A.A. (2011). Progress in starch modification in the last decade. Food Hydrocoll..

[31] Dewi A.M.P., Santoso U., Pranoto Y., Marseno D.W. (2022). Dual Modification of Sago Starch via Heat Moisture Treatment and Octenyl Succinylation to Improve Starch Hydrophobicity. Polymers.

[32] Pech-Cohuo S.C., Martín-López H., Uribe-Calderón J., González-Canché N.G., Salgado-Tránsito I., May-Pat A., Cuevas-Bernardino J.C., Ayora-Talavera T., Cervantes-Uc J.M., Pacheco N. (2022). Physicochemical, Mechanical, and Structural Properties of Bio-Active Films Based on Biological-Chemical Chitosan, a Novel Ramon (*Brosimum alicastrum*) Starch, and Quercetin. Polymers.

[33] da Silva L.R., Velasco J.I., Fakhouri F.M. (2022). Bioactive Films Based on Starch from White, Red, and Black Rice to Food Application. Polymers.

[34] Rodrigues G.D.M., Filgueiras C.T., Garcia V.A.D.S., De Carvalho R.A., Velasco J.I., Fakhouri F.M. (2020). Antimicrobial Activity and GC-MS Profile of Copaiba Oil for Incorporation into *Xanthosoma mafaffa* Schott Starch-Based Films. Polymers.

[35] Aliabadi M., Chee B.S., Matos M., Cortese Y.J., Nugent M.J.D., De Lima T.A.M., Magalhães W.L.E., De Lima G.G. (2020). Yerba Mate Extract in Microfibrillated Cellulose and Corn Starch Films as a Potential Wound Healing Bandage. Polymers.

[36] Jiang F., Du C., Zhao N., Jiang W., Yu X., Du S.-K. (2021). Preparation and characterization of quinoa starch nanoparticles as quercetin carriers. Food Chem..

[37] Mudgil P., Aldhaheri F., Hamdi M., Punia S., Maqsood S. (2022). Fortification of Chami (traditional soft cheese) with probiotic-loaded protein and starch microparticles: Characterization, bioactive properties, and storage stability. LWT.

[38] Jamroży M., Głąb M., Kudłacik-Kramarczyk S., Drabczyk A., Gajda P., Tyliszczak B. (2022). The Impact of the *Matricaria chamomilla* L. Extract, Starch Solution and the Photoinitiator on Physiochemical Properties of Acrylic Hydrogels. Materials.

[39] Wang P., Luo Z.-G., Xiao Z.-G. (2021). Preparation, physicochemical characterization and in vitro release behavior of resveratrol-loaded oxidized gellan gum/resistant starch hydrogel beads. Carbohydr. Polym..

[40] Elvira C., Mano J.F., San J., An R., Reis R.L. (2002). Starch-Based Biodegradable Hydrogels with Potential Biomedical Applications as Drug Delivery Systems. Biomaterials.

[41] Mun S., Kim Y.-R., McClements D.J. (2014). Control of β-carotene bioaccessibility using starch-based filled hydrogels. Food Chem..

[42] Xiao C. (2012). Current advances of chemical and physical starch-based hydrogels. Starch-Stärke.

[43] Leites Luchese C., Fernando L., Brum W., Piovesana A., Caetano K., Flôres S.H. (2017). Bioactive Compounds Incorporation into the Production of Functional Biodegradable Films—A Review. Polym. Renew. Resour..

[44] Petrovska B.B. (2012). Historical Review of Medicinal Plants’ Usage. Pharmacogn. Rev..

[45] Dias D.A., Urban S., Roessner U. (2012). A Historical Overview of Natural Products in Drug Discovery. Metabolites.

[46] Kris-Etherton P.M., Hecker K.D., Bonanome A., Coval S.M., Binkoski A.E., Hilpert K.F., Griel A.E., Etherton T.D. (2002). Bioactive compounds in foods: Their role in the prevention of cardiovascular disease and cancer. Am. J. Med..

[47] Chandra H., Bishnoi P., Yadav A., Patni B., Mishra A.P., Nautiyal A.R. (2017). Antimicrobial Resistance and the Alternative Re-sources with Special Emphasis on Plant-Based Antimicrobials—A Review. Plants.

[48] Maeda H.A. (2019). Evolutionary Diversification of Primary Metabolism and Its Contribution to Plant Chemical Diversity. Front. Plant Sci..

[49] Azmir J., Zaidul I.S.M., Rahman M.M., Sharif K.M., Mohamed A., Sahena F., Jahurul M.H.A., Ghafoor K., Norulaini N.A.N., Omar A.K.M. (2013). Techniques for extraction of bioactive compounds from plant materials: A review. J. Food Eng..

[50] Fang C., Fernie A.R., Luo J. (2018). Exploring the Diversity of Plant Metabolism. Trends Plant Sci..

[51] Rhodes M.J.C. (1994). Mini-Review Physiological Roles for Secondary Metabolites in Plants: Some Progress, Many Outstanding Problems. Plant Mol. Biol..

[52] Jafarzadeh S., Jafari S.M., Salehabadi A., Nafchi A.M., Kumar U.S.U., Khalil H.A. (2020). Biodegradable green packaging with antimicrobial functions based on the bioactive compounds from tropical plants and their by-products. Trends Food Sci. Technol..

[53] Mahcene Z., Khelil A., Hasni S., Akman P.K., Bozkurt F., Birech K., Goudjil M.B., Tornuk F. (2019). Development and characterization of sodium alginate based active edible films incorporated with essential oils of some medicinal plants. Int. J. Biol. Macromol..

[54] Brito T., Carrajola J., Gonçalves E., Martelli-Tosi M., Ferreira M. (2019). Fruit and vegetable residues flours with different granulometry range as raw material for pectin-enriched biodegradable film preparation. Food Res. Int..

[55] Othman L., Sleiman A., Abdel-Massih R.M. (2019). Antimicrobial Activity of Polyphenols and Alkaloids in Middle Eastern Plants. Front. Microbiol..

[56] Cragg G.M., Newman D.J. (2013). Natural products: A continuing source of novel drug leads. Biochim. Biophys. Acta BBA Gen. Subj..

[57] Alencar Pamphile J., Costa A.T., Rosseto P., Polonio J.C., Pereira J.O., Azevedo J.L. (2017). Aplicações biotecnológicas de metabólitos secundários extraídos de fungos endofíticos: O caso do Colletotrichum sp. Biotechnological applications of sec-ondary metabolites extracted from endophytic fungi: The case of Colletotrichum Sp.. Rev. Uningá.

[58] Newman D.J., Cragg G.M. (2020). Natural Products as Sources of New Drugs over the Nearly Four Decades from 01/1981 to 09/2019. J. Nat. Prod..

[59] Panyoyai N., Shanks R., Kasapis S. (2017). Tocopheryl acetate release from microcapsules of waxy maize starch. Carbohydr. Polym..

[60] Jung Y.-S., Hong M.-G., Park S.-H., Lee B.-H., Yoo S.-H. (2019). Biocatalytic Fabrication of α-Glucan-Coated Porous Starch Granules by Amylolytic and Glucan-Synthesizing Enzymes as a Target-Specific Delivery Carrier. Biomacromolecules.

[61] Athira G.K., Jyothi A.N., Vishnu V.R. (2018). Water Soluble Octenyl Succinylated Cassava Starch-Curcumin Nanoformulation With Enhanced Bioavailability and Anticancer Potential. Starch-Stärke.

[62] Luo K., Adra H.J., Kim Y.-R. (2020). Preparation of starch-based drug delivery system through the self-assembly of short chain glucans and control of its release property. Carbohydr. Polym..

[63] Lu X., Li C., Huang Q. (2019). Combining in vitro digestion model with cell culture model: Assessment of encapsulation and delivery of curcumin in milled starch particle stabilized Pickering emulsions. Int. J. Biol. Macromol..

[64] Schaefer E.W., Pavoni J.M.F., Luchese C.L., Faccin D.J.L., Tessaro I.C. (2020). Influence of turmeric incorporation on physicochemical, antimicrobial and mechanical properties of the cornstarch and chitosan films. Int. J. Biol. Macromol..

[65] Oliyaei N., Moosavi-Nasab M., Tamaddon A.M., Tanideh N. (2021). Antidiabetic effect of fucoxanthin extracted from Sargassum angustifolium on streptozotocin-nicotinamide-induced type 2 diabetic mice. Food Sci. Nutr..

[66] Costa N.N., Lopes L.D.F., Ferreira D.F., de Prado E.M.L., Severi J.A., Resende J.A., Careta F.D.P., Ferreira M.C.P., Carreira L.G., de Souza S.O.L. (2020). Polymeric films containing pomegranate peel extract based on PVA/starch/PAA blends for use as wound dressing: In vitro analysis and physicochemical evaluation. Mater. Sci. Eng. C.

[67] Garcia V.A.D.S., Borges J.G., Mazalli M.R., Lapa-Guimarães J.D.G., Vanin F.M., De Carvalho R.A. (2017). Gelatin and pregelatinized starch orally disintegrating films: Properties and stability of vitamin C. J. Appl. Polym. Sci..

[68] Garcia V.A.D.S., Borges J.G., Maciel V., Mazalli M.R., Lapa-Guimaraes J.D.G., Vanin F.M., de Carvalho R.A. (2018). Gelatin/starch orally disintegrating films as a promising system for vitamin C delivery. Food Hydrocoll..

[69] Guerra A.P., Bitencourt Cervi C.M., Dos V.A., Garcia S., da Silva C. (2019). Incorporation of active compounds from mango peel (*Mangifera indica* L. Cv. “tommy atkins”) into corn starch-based oral disintegrating films. Lat. Am. Appl. Res..

[70] Bodini R.B., Pugine S.M.P., de Melo M.P., de Carvalho R.A. (2020). Antioxidant and anti-inflammatory properties of orally disintegrating films based on starch and hydroxypropyl methylcellulose incorporated with Cordia verbenacea (erva baleeira) extract. Int. J. Biol. Macromol..

[71] Pelissari F.M., Grossmann M.V.E., Yamashita F., Pineda E.A.G. (2009). Antimicrobial, Mechanical, and Barrier Properties of Cassava Starch−Chitosan Films Incorporated with Oregano Essential Oil. J. Agric. Food Chem..

[72] De Araújo G.K.P., De Souza S.J., Da Silva M.V., Yamashita F., Gonçalves O.H., Leimann F.V., Shirai M.A. (2015). Physical, antimicrobial and antioxidant properties of starch-based film containing ethanolic propolis extract. Int. J. Food Sci. Technol..

[73] Taweechat C., Wongsooka T., Rawdkuen S. (2021). Properties of Banana (*Cavendish* spp.) Starch Film Incorporated with Banana Peel Extract and Its Application. Molecules.

[74] Boeira C.P., Alves J.D.S., Flores D.C.B., de Moura M.R., Melo P.T.S., da Rosa C.S. (2021). Antioxidant and antimicrobial effect of an innovative active film containing corn stigma residue extract for refrigerated meat conservation. J. Food Process. Preserv..

[75] Malherbi N.M., Schmitz A.C., Grando R.C., Bilck A.P., Yamashita F., Tormen L., Fakhouri F.M., Velasco J.I., Bertan L.C. (2018). Corn starch and gelatin-based films added with guabiroba pulp for application in food packaging. Food Packag. Shelf Life.

[76] Tongdeesoontorn W., Mauer L., Wongruong S., Sriburi P., Reungsang A., Rachtanapun P. (2021). Antioxidant Films from Cassava Starch/Gelatin Biocomposite Fortified with Quercetin and TBHQ and Their Applications in Food Models. Polymers.

[77] Peralta J., Bitencourt-Cervi C.M., Maciel V., Yoshida C.M., Carvalho R.A. (2018). Aqueous hibiscus extract as a potential natural pH indicator incorporated in natural polymeric films. Food Packag. Shelf Life.

[78] da Silva T.B.V., Moreira T.F.M., de Oliveira A., Bilck A.P., Gonçalves O.H., Ferreira I.C.F.R., Barros L., Barreiro M.-F., Yamashita F., Shirai M.A. (2019). *Araucaria angustifolia* (Bertol.) Kuntze extract as a source of phenolic compounds in TPS/PBAT active films. Food Funct..

[79] Müller P.S., Carpiné D., Yamashita F., Waszczynskyj N. (2020). Influence of pinhão starch and natural extracts on the performance of thermoplastic cassava starch/PBAT extruded blown films as a technological approach for bio-based packaging material. J. Food Sci..

[80] de Oliveira T.V., de Freitas P.A.V., Pola C.C., da Silva J.O.R., Diaz L.D.A., Ferreira S.O., Soares N.D.F. (2020). Development and optimization of antimicrobial active films produced with a reinforced and compatibilized biodegradable polymers. Food Packag. Shelf Life.

[81] Souza P.N.D.C., Tavares D.G., Souza C.R.F., Martinez M.L.L., Oliveira W.P., Guimarães L.H.S., Cardoso P.G. (2020). Spray Drying of Coloring Extracts Produced by Fungi Isolated from Brazilian Caves. Braz. Arch. Biol. Technol..

[82] Ribeiro A.M., Shahgol M., Estevinho B.N., Rocha F. (2020). Microencapsulation of Vitamin A by spray-drying, using binary and ternary blends of gum arabic, starch and maltodextrin. Food Hydrocoll..

[83] Tomsone L., Galoburda R., Kruma Z., Durrieu V., Cinkmanis I. (2020). Microencapsulation of Horseradish (*Armoracia rusticana* L.) Juice Using Spray-Drying. Foods.

[84] Abbas S., Bashari M., Akhtar W., Li W.W., Zhang X. (2014). Process optimization of ultrasound-assisted curcumin nanoemulsions stabilized by OSA-modified starch. Ultrason. Sonochem..

[85] Doost A.S., Afghari N., Abbasi H., Nasrabadi M.N., Dewettinck K., Van der Meeren P. (2020). Nano-lipid carriers stabilized by hydrophobically modified starch or sucrose stearate for the delivery of lutein as a nutraceutical beverage model. Colloids Surfaces A Physicochem. Eng. Asp..

[86] Niu Z., Acevedo-Fani A., McDowell A., Barnett A., Loveday S.M., Singh H. (2020). Nanoemulsion structure and food matrix determine the gastrointestinal fate and in vivo bioavailability of coenzyme Q10. J. Control. Release.

[87] Hasani S., Ojagh S.M., Ghorbani M. (2018). Nanoencapsulation of lemon essential oil in Chitosan-Hicap system. Part 1: Study on its physical and structural characteristics. Int. J. Biol. Macromol..

[88] Silva R.S., Santos C.D.L., Mar J.M., Kluczkovski A.M., Figueiredo J.D.A., Borges S.V., Bakry A.M., Sanches E.A., Campelo P.H. (2018). Physicochemical properties of tucumã (*Astrocaryum aculeatum*) powders with different carbohydrate biopolymers. LWT.

[89] Ravichai K., Muangrat R. (2019). Effect of different coating materials on freeze-drying encapsulation of bioactive compounds from fermented tea leaf wastewater. J. Food Process. Preserv..

[90] Zheng B., Xie F., Situ W., Chen L., Li X. (2017). Controlled bioactive compound delivery systems based on double polysaccharide film-coated microparticles for liquid products and their release behaviors. J. Funct. Foods.

[91] Wang X., Li X., Chen L., Xie F., Yu L., Li B. (2010). Preparation and characterisation of octenyl succinate starch as a delivery carrier for bioactive food components. Food Chem..

[92] Rodrigues R., Bilibio D., Plata-Oviedo M.S.V., Pereira E.A., Mitterer-Daltoé M.L., Perin E.C., Carpes S.T. (2021). Microencapsulated and Lyophilized Propolis Co-Product Extract as Antioxidant Synthetic Replacer on Traditional Brazilian Starch Biscuit. Molecules.

[93] Daudt R.M., Back P.I., Cardozo N., Marczak L.D.F., Külkamp-Guerreiro I. (2015). Pinhão starch and coat extract as new natural cosmetic ingredients: Topical formulation stability and sensory analysis. Carbohydr. Polym..

[94] Silveira M.P., Silva H.C., Pimentel I.C., Poitevin C.G., Stuart A.K.D.C., Carpiné D., Jorge L.M.D.M., Jorge R.M.M. (2019). Development of active cassava starch cellulose nanofiber-based films incorporated with natural antimicrobial tea tree essential oil. J. Appl. Polym. Sci..

[95] Wu D., Samanta A., Srivastava R.K., Hakkarainen M. (2017). Starch-Derived Nanographene Oxide Paves the Way for Electrospinnable and Bioactive Starch Scaffolds for Bone Tissue Engineering. Biomacromolecules.

[96] Hornung P.S., Ávila S., Apea-Bah F.B., Liu J., Teixeira G.L., Ribani R.H., Beta T. (2020). Sustainable Use of Ilex paraguariensis Waste in Improving Biodegradable Corn Starch Films’ Mechanical, Thermal and Bioactive Properties. J. Polym. Environ..

[97] Pereira J.F., Lonni A.A.S.G., Mali S. (2021). Development of biopolymeric films with addition of vitamin C and catuaba extract as natural antioxidants. Prep. Biochem. Biotechnol..

[98] Park K. (2014). Controlled drug delivery systems: Past forward and future back. J. Control. Release.

[99] Tran T.T.D., Tran P.H.L. (2019). Controlled Release Film Forming Systems in Drug Delivery: The Potential for Efficient Drug De-livery. Pharmaceutics.

[100] Pachuau L. (2015). Recent developments in novel drug delivery systems for wound healing. Expert Opin. Drug Deliv..

[101] Pacheco M.S., Barbieri D., da Silva C.F., de Moraes M.A. (2021). A Review on Orally Disintegrating Films (ODFs) Made from Natural Polymers Such as Pullulan, Maltodextrin, Starch, and Others. Int. J. Biol. Macromol..

[102] Luzi F., Torre L., Kenny J.M., Puglia D. (2019). Bio- and Fossil-Based Polymeric Blends and Nanocomposites for Packaging: Structure–Property Relationship. Materials.

[103] Peelman N., Ragaert P., De Meulenaer B., Adons D., Peeters R., Cardon L., Van Impe F., Devlieghere F. (2013). Application of bioplastics for food packaging. Trends Food Sci. Technol..

[104] de Léis C.M., Nogueira A.R., Kulay L., Tadini C. (2017). Environmental and energy analysis of biopolymer film based on cassava starch in Brazil. J. Clean. Prod..

[105] Taherimehr M., YousefniaPasha H., Tabatabaeekoloor R., Pesaranhajiabbas E. (2021). Trends and challenges of biopolymer-based nanocomposites in food packaging. Compr. Rev. Food Sci. Food Saf..

[106] Ribeiro J.S., Veloso C.M. (2020). Microencapsulation of natural dyes with biopolymers for application in food: A review. Food Hydrocoll..

[107] Mitura S., Sionkowska A., Jaiswal A. (2020). Biopolymers for Hydrogels in Cosmetics: Review. J. Mater. Sci. Mater. Med..

